# A phase 3 study of the efficacy and safety of avatrombopag in Japanese adults with chronic immune thrombocytopenia

**DOI:** 10.1007/s12185-025-04001-4

**Published:** 2025-05-20

**Authors:** Hiroki Yamaguchi, Masaki Iino, Shugo Kowata, Ryusuke Yamamoto, Jun Yamanouchi, Yutaka Imamura, Keita Kirito, Kenji Yokoyama, Tomoki Ito, Tatsunori Ishikawa, Motoaki Shiratsuchi, Yoshiaki Tomiyama, Harumi Kamiya, Jessica Zhang, Brian D. Jamieson

**Affiliations:** 1 Department of Hematology, Nippon Medical School Hospital, Tokyo, Japan; 2 Department of Hematology and Hematopoietic Stem Cell Transplantation, Yamanashi Prefectural Central Hospital, Yamanashi, Japan; 3 Division of Hematology and Oncology, Department of Internal Medicine, Iwate Medical University, Iwate, Japan; 4 Department of Hematology, Kobe City Medical Center General Hospital, Hyogo, Japan; 5 Division of Blood Transfusion and Cell Therapy, Ehime University Hospital, Ehime, Japan; 6 Division of Hematology, St. Mary’s Hospital, Fukuoka, Japan; 7 University of Yamanashi, Yamanashi, Japan; 8 Tokai University Hachioji Hospital, Tokyo, Japan; 9 Kansai Medical University, Osaka, Japan; 10 Chugoku Central Hospital, Hiroshima, Japan; 11 Aso Iizuka Hospital, Fukuoka, Japan; 12 Department of Blood Transfusion, Osaka University Hospital, Osaka, Japan; 13 Sobi, K.K., Tokyo, Japan; 14 Sobi, Inc, Morrisville, NC USA

**Keywords:** Clinical trial, ITP, Japan, Thrombopoietin receptor agonist

## Abstract

Avatrombopag is an oral thrombopoietin receptor agonist approved widely for the treatment of adults with chronic immune thrombocytopenia (ITP). However, data in Japanese patients are limited. This confirmatory phase 3, open-label study investigated avatrombopag (initial dose 20 mg/day) in Japanese adults (aged ≥ 18 years) with chronic ITP (≥ 12 months), insufficient response to prior treatment and an average of 2 platelet counts (PCs) < 30 × 10^9^/L. The primary endpoint was the cumulative number of weeks of platelet response (PC ≥ 50 × 10^9^/L) without rescue therapy for bleeding. Secondary endpoints included platelet response rate at Day 8 and safety. In total, 19 patients were enrolled into the 26-week core phase. The mean age was 56.0 years; 78.9% of patients were female. Mean cumulative number of weeks of platelet response was 13.5 (95% CI 9.1–17.8). Platelet response at Day 8 was achieved by 63.2% of patients. In the core phase, adverse events (AEs) occurred in 94.7% of patients and serious AEs in 15.8%. No thromboembolic events or deaths occurred in the core phase. Avatrombopag demonstrated a rapid and durable platelet response and was well tolerated in Japanese patients with chronic ITP. Long-term safety and efficacy are being assessed in the ongoing extension phase.

**Supplementary Information:**

The online version contains supplementary material available at 10.1007/s12185-025-04001-4.

## Introduction

Immune thrombocytopenia (ITP) is a rare, acquired autoimmune disorder which can affect both adults and children and is characterised by low platelet counts (PCs) due to impaired production and increased destruction of platelets [1, 2]. The incidence of ITP in adults in Japan between 2004 and 2007 was 2.20 per 100,000/year, with a higher incidence reported in women (2.58 per 100,000/year) compared with men (1.72 per 100,000/year) [3]. This is similar to global incidence estimates of 3.3 per 100,000/year [4].

Symptoms of ITP include bruising, epistaxis, bleeding gums, extra bleeding during surgery and, more rarely, severe mucosal bleeding events, menorrhagia and blood in urine/stool [2, 5, 6]. In addition, ITP significantly affects health-related quality of life, with many patients experiencing fatigue and anxiety or depression [5]. Therefore, treatment goals for people with ITP are to maintain a safe PC (≥ 30 × 10^9^/L) to prevent severe bleeding and ensure acceptable health-related quality of life [1, 6, 7].

In Japan, guidelines recommend first-line treatment of ITP with corticosteroids [7]. However, corticosteroids are associated with variable and transient efficacy, as well as short- and long-term adverse events (AEs) [7, 8]. In a summary of 12 case series spanning more than 6 decades (1928–1989), only 26% of patients with ITP treated with corticosteroids achieved complete response [9]. Japanese guidelines recommend switching to second-line or third-line treatment if the therapeutic target is not reached (the patient is unable to maintain a PC sufficient to prevent serious bleeding, usually ≥ 30 × 10^9^/L) [7]. Recommended second-line treatments are thrombopoietin receptor agonists (TPO-RAs), rituximab or splenectomy [7].

The TPO-RAs, eltrombopag and romiplostim, are generally effective and well-tolerated in adult patients who have chronic ITP [10–14]. In Japan, eltrombopag was approved for chronic ITP in 2010, and romiplostim in 2011 [15, 16]. However, there are some notable treatment challenges associated with both eltrombopag and romiplostim. Firstly, regarding efficacy, loss of treatment response is a common challenge with TPO-RA use, having been reported as the most common reason for switching between eltrombopag and romiplostim in a real-world UK study [17]. Secondly, regarding safety, eltrombopag is associated with risk of hepatic decompensation in patients with chronic hepatitis C and can cause abnormal liver function and severe hepatotoxicity, which might be life-threatening; as such, liver monitoring is necessary for all patients, as described in the box warning [18, 19]. Finally, there are challenges associated with routes of administration for romiplostim and eltrombopag. Romiplostim is administered by subcutaneous injection, whereas patients with ITP prefer oral administration [20–23]. Although eltrombopag is orally administered, food restrictions are required, whereas people with ITP prefer treatments without food restrictions [20–22].

Avatrombopag, an oral TPO-RA, has been studied in healthy adults in 2 single- and multiple-dose phase 1 dose-rising, safety and tolerability studies [24], and in patients with persistent (3–12 months [1]) and chronic (> 12 months [1]) ITP in a phase 2 randomised study [25]. Efficacy and safety of avatrombopag were also evaluated in 2 pivotal phase 3 studies (one global, the other in China) in adult patients with chronic ITP and a PC < 30 × 10^9^/L [26, 27]. In the USA, avatrombopag was approved in 2018 for the treatment of thrombocytopenia in adult patients with chronic liver disease undergoing an elective procedure [28]. It was subsequently approved for the treatment of primary chronic ITP in adult patients who are refractory to other treatments (e.g., corticosteroids, immunoglobulins) in 2019 [28] and in the EU in 2021 [29]. Avatrombopag was approved for chronic liver disease in patients undergoing an elective procedure in China in 2020 [30, 31] and in Japan in 2023 [32]. Unlike eltrombopag, avatrombopag has no significant hepatotoxicity, no requirement for additional liver monitoring and it is not affected by food type or supplements, so there are no restrictions on meal composition [18, 19, 28, 29]. Approximately 88% of an administered avatrombopag dose is excreted faecally and 6% renally [33]. It has been established that avatrombopag does not have the potential to chelate cations or iron [28].

In a randomised, open-label single-dose study, there were no clinically relevant differences in avatrombopag pharmacokinetics (PK) or pharmacodynamics (PD) between Japanese and White healthy adults [33]. However, to date, there are limited data available regarding the efficacy and safety of avatrombopag in Japanese patients with ITP.

This study was conducted to confirm that there were no efficacy, safety or PK differences between Japanese patients treated with avatrombopag and patients from other global and national studies treated with avatrombopag. The study had an open-label study design without a placebo group, given that the mean number of cumulative weeks with a platelet response was zero in the placebo group in the pivotal phase 3 study [26].

## Methods

### Study design

AVA-ITP-307 was a phase 3, open-label study conducted across 19 centres in Japan. The study was registered with ClinicalTrials.gov (NCT05369208) and the Japan Registry of Clinical Trials (jRCT2031220005).

### Patients

Eligible patients were aged ≥ 18 years with chronic ITP (≥ 12 months), had insufficient response to prior treatment for ITP (investigator assessed) and an average of 2 PCs < 30×10^9^/L (no single PC could be > 35×10^9^/L). The 2 samples must have been obtained ≥ 48 h and ≤ 2 weeks apart.

Key exclusion criteria included known secondary ITP, known inherited thrombocytopenia, myelodysplastic syndrome, arterial or venous thrombosis, significant cardiovascular disease, cirrhosis, portal hypertension, chronic active hepatitis, malignant disease and use of immunoglobulins, corticosteroids, splenectomy or rituximab within 12 weeks of Day 1, romiplostim or eltrombopag within 1 week of Day 1.

### Procedures

**Fig. 1:**
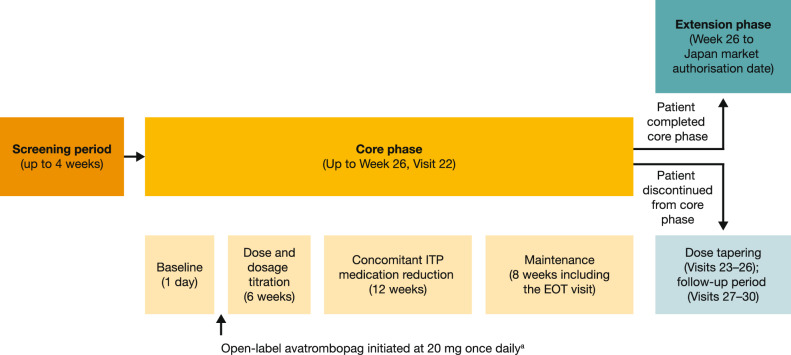
Study design. ^a^Avatrombopag starting dose was 20 mg once daily. Avatrombopag dose was titrated up to a maximum dose of 40 mg once daily or down to a minimum dose of 20 mg once weekly, according to pre-specified PC thresholds. *EOT* end of treatment, *ITP* immune thrombocytopenic purpura, *PC* platelet count

Following an initial screening period of up to 4 weeks, the study had a 26-week core phase (which is completed) and a subsequent extension phase, which is currently ongoing (Fig. 1). The core phase included a 1-day baseline assessment, a 6-week dosage titration phase, a 12-week concomitant medication reduction phase and, finally, an 8-week maintenance phase.

In the dosage titration phase, patients received avatrombopag at a starting dose of oral 20 mg once daily with food. The dose was titrated up to a maximum dose of 40 mg once daily or down to 20 mg once weekly, according to individual response to treatment (target PC 50 to < 200 × 10^9^/L), and in accordance with approved US Food and Drug Administration dose levels for titration (Supplementary Table S1) [28]. Downward titration of concomitant ITP medication was not permitted during this phase unless there was a safety concern.

During the concomitant ITP medication reduction period, downward titration of concomitant ITP medication was permitted at the investigator’s discretion, but should only be considered if the patient’s PC remained above 200 × 10^9^/L.

During the maintenance period, patients continued avatrombopag treatment with dosage adjustments made in accordance with approved overseas labelling [28], to maintain a PC of 50 to < 200 × 10^9^/L. Downward titration of concomitant ITP medication was not permitted during this phase unless there was a safety concern.

If a patient’s PC was ≥ 200 to ≤ 400 × 10^9^/L, avatrombopag dose was decreased one level, followed by a 2-week wait to assess the dose effects. If a patient’s PC was > 400 × 10^9^/L, avatrombopag was discontinued and platelet monitoring was increased to twice weekly. Avatrombopag was also discontinued if PC was < 50 × 10^9^/L after 4 weeks of 40 mg dose once daily or if PC was > 400 × 10^9^/L after 2 weeks of 20 mg dose once weekly [28]. Full dose adjustment guidelines are available in the US Prescribing Information [28].

Permitted ITP concomitant medications included corticosteroids and/or azathioprine, taken at a stable dose for 4 weeks before enrolment; and mycophenolate mofetil, cyclosporin A or danazol, taken at a stable dose for at least 12 weeks before enrolment.

### Core phase efficacy endpoints

The primary endpoint was the cumulative number of weeks during which PC was ≥ 50×10^9^/L, in the absence of rescue therapy. The pre-defined threshold for achieving the primary efficacy endpoint was that the lower limit of the 95% confidence interval (CI) of the mean number of cumulative weeks of platelet response ≥ 50×10^9^/L was ≥ 8.02 weeks. This was defined based on a study of eltrombopag in Japanese adults with ITP [10, 26]. The key secondary endpoint was the platelet response rate at Day 8 (percentage of patients with a PC ≥ 50×10^9^/L), in the absence of rescue therapy.

Other efficacy assessments included: durable platelet response (6 out of 8 weekly PCs ≥ 50 × 10^9^/L during the last 8 weeks of treatment over the 26-week core phase treatment period, in the absence of rescue therapy); maximum duration of continuous platelet response (the number of consecutive weeks with PC ≥ 50 × 10^9^/L, in the absence of rescue therapy); International Working Group (IWG) complete platelet response (PC ≥ 100 × 10^9^/L and absence of bleeding or rescue therapy at each analysis visit); IWG platelet response (PC ≥ 30 × 10^9^/L, at least a twofold increase in baseline PC and absence of bleeding or rescue therapy at each analysis visit); median PC by visit (target range of 50–200 × 10^9^/L during the core phase from Week 1 to Week 26); use of rescue therapy, reduction/discontinuation of concomitant ITP treatment from baseline; and bleeding symptoms (assessed by World Health Organization [WHO] Bleeding scale).

### Core phase safety endpoints

Safety was assessed by the investigator and included treatment-emergent AEs (TEAEs), serious AEs (SAEs), AEs of special interest (AESIs; thromboembolic events [TEEs] and WHO Grade 3 or 4 bleeding events), clinical laboratory assessments, vital signs and physical examinations.

### Core phase PK endpoints

Population PKs were assessed using plasma concentrations of avatrombopag. Blood samples for serial PK assessments were collected during Week 2 (Visit 5) and Week 10 (Visit 10) of the core phase. Blood samples for sparse PK assessments were collected during Week 1 (Visit 4), Week 4 (Visit 7), Week 6 (Visit 8), Week 16 (Visit 13) and Week 26 (Visit 22) of the core phase.

### Extension phase assessments

In the ongoing extension phase, efficacy is being evaluated by median PC, proportion of patients needing rescue therapy, and incidence and severity of bleeding events. Safety is also being evaluated as per the core phase. Efficacy and safety data are being collected monthly and will be reported in a future publication.

### Statistical analyses

The full analysis set included all patients who were enrolled into the study. The per-protocol set included all patients who received the protocol-assigned study drug and who did not meet any pre-specified criteria. The safety analysis set included all patients who received at least one dose of study drug and had a post-dose safety assessment.

The evaluation of efficacy was performed using the full analysis set. Subgroup analyses for the primary efficacy endpoint were conducted. The subgroups were defined as age (< 65 years, ≥ 65 years), sex (male, female), baseline PC (≤ 15 × 10^9^/L, > 15 × 10^9^/L), use of concomitant ITP medications (yes, no), splenectomy status (yes, no) and baseline creatinine clearance (≤ 30 to  < 60 mL/min, ≤ 60 to  < 90 mL/min, ≥ 90 mL/min, missing).

The evaluation of safety was performed using the safety analysis set. All AEs were coded to System Organ Class and preferred term using Medical Dictionary for Regulatory Activities version 26.1.

Descriptive statistics of avatrombopag plasma concentrations by analysis visit/time points in the safety analysis set were calculated. Due to the small number of patients in this study, MAXEVAL = 0 and model validation were used to evaluate if PK and PD in Japanese patients differed from ‘legacy patients’ using previously developed population PK and population PK/PD models. The population PK and population PK/PD datasets included ‘legacy patients’ from phase 1 studies (healthy patients; included in population PK dataset but not the population PK/PD dataset), and phase 2 and phase 3 studies (chemotherapy-induced thrombocytopenia [CIT] for the popPK dataset and patients with chronic liver disease [CLD], ITP or CIT for the popPK/PD dataset).

### Role of the funding source

The sponsor of the study was involved in the study design and monitoring, data collection, data analysis and data interpretation. Medical writing and editorial support were funded by the study sponsor.

The protocol for this study was reviewed by institutional review boards for each investigational site. The study was conducted in compliance with the protocol and International Conference on Harmonisation of Technical Requirements for Registration of Pharmaceuticals for Human Use, the provisions of the Declaration of Helsinki, Good Clinical Practice guidelines and all local laws and regulations. All patients provided written informed consent before commencement of any trial-related activities.

## Results

### Patients

**Fig. 2:**
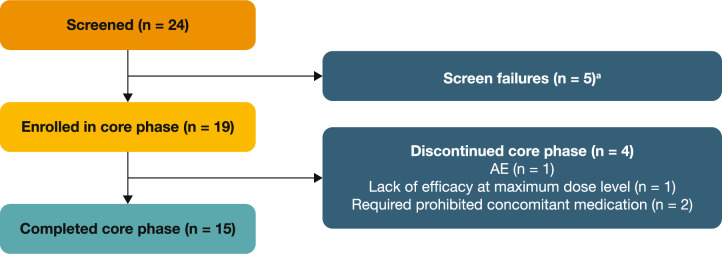
Patient disposition in the 26-week core phase (full analysis set). ^a^Five patients failed to meet the inclusion criterion ‘Patients have an average of 2 platelet counts < 30 × 10^9^/L (no single count can be 35 × 10^9^/L). The 2 samples must be obtained ≥ 48 h and ≤ 2 weeks apart’

In total, 19 adult patients were enrolled in the core phase of the study, which was conducted between 8 June 2022 and 17 January 2024 (data cut-off date). Four patients discontinued the core phase (due to AEs [n = 1], lack of efficacy at the highest dose [n = 1] and use of concomitant medication [n = 2]); 15 patients completed the core phase (Fig. 2). The extension phase of the study is currently ongoing, with all 15 patients who completed the core phase having been enrolled.

The majority of the patients were female (78.9%), and the mean age was 56.0 years (Table 1). Eight patients had a baseline PC ≤ 15 × 10^9^/L, and 9 patients used concomitant ITP medication at baseline. Three patients had at least one previous significant bleeding event prior to the core phase. Two patients had been splenectomised prior to the study. Of relevance, 15 patients had a history of prior TPO-RA use. Fifteen patients had previously taken eltrombopag, 2 of which had also previously used romiplostim.

**Table 1:** Demographics and baseline clinical characteristics (safety analysis set)

Characteristic	Avatrombopag (N = 19)
Age, median (min, max), years	60.0 (19, 74)
< 65 years	12 (63.2)
Sex	
Male, n (%)	4 (21.1)
Female, n (%)	15 (78.9)
Race, n (%)	
Japanese	19 (100.0)
Height, median (min, max), cm	157.0 (144, 179)
Weight, median (min, max), kg	61.70 (43.4, 81.8)
BMI, median (min, max), kg/m^2^	23.89 (17.6, 32.4)
Use of concomitant ITP medication at baseline^a^, n (%)	9 (47.4)
Previous treatment for ITP, n (%)	19 (100)
Eltrombopag	15 (78.9)
Romiplostim^b^	2 (10.5)
Rituximab	4 (21.1)
Immunosuppressant	15 (78.9)
Immunoglobulins	5 (26.3)
Splenectomy, n (%)	2 (10.5)
Number of platelet transfusions in the previous 1 year^c^, n (%)	
0	16 (84.2)
1	1 (5.3)
2	1 (5.3)
3	1 (5.3)
Number of previous hospitalisations for ITP^c^, n (%)	
0	10 (52.6)
1	4 (21.1)
2	3 (15.8)
3	1 (5.3)
4	1 (5.3)
Number of previous significant bleeding events^c^, n (%)	
0	16 (84.2)
1	2 (10.5)
2	0
3	1 (5.3)
Baseline platelet count, n (%)	
≤ 15 × 10^9^/L	8 (42.1)
> 15 × 10^9^/L	11 (57.9)
Baseline creatinine clearance, n (%)	
30 to < 60 mL/min	2 (10.5)
60 to < 90 mL/min	8 (42.1)
≥ 90 mL/min	9 (47.4)

*BMI* body mass index, *ITP* immune thrombocytopenic purpura

^a^Permitted ITP concomitant medications included corticosteroids and/or azathioprine, taken at a stable dose for 4 weeks before enrolment; and mycophenolate mofetil, cyclosporin A or danazol, taken at a stable dose for at least 12 weeks before enrolment

^b^Two patients had previously used romiplostim, as well as having previously used eltrombopag

^c^Recorded during patient’s ITP history

### Efficacy

Platelet response efficacy data are presented in Table 2. During the 26-week core phase, the mean cumulative number of weeks in which patients had PC ≥ 50×10^9^/L, in the absence of rescue therapy (primary endpoint), was 13.5 weeks (95% CI 9.1–17.8; standard deviation [SD] 9.0). The lower limit of the 95% CI was higher than the threshold of ≥ 8.02 weeks, indicating the primary endpoint was achieved. The percentage of patients with a platelet response (PC ≥ 50×10^9^/L) at Visit 4 (Day 8) (key secondary endpoint) was 63.2% (95% CI 38.4–83.7).

**Table 2:** Efficacy: platelet response endpoints (full analysis set)

	Avatrombopag (N = 19)
Primary endpoint: cumulative number of weeks of platelet response^a^	
Mean (SD) [95% CI] Median (IQR)	13.5 (9.0) [9.1–17.8] 16.6 (3.0–20.4)
Platelet response rate at Visit 4 (Day 8)^b^, n (%) [95% CI]	12 (63.2) [38.4–83.7]
Durable platelet response^c^, n (%) [95% CI]	8 (42.1) [20.3–66.5]
Maximum duration (weeks) of platelet response^d^	
Mean (SD) Median (IQR)	7.6 (6.9) 7.0 (2.0–13.0)
Complete response by IWG definition^e^, n (%)	
By Visit 4 (Day 8) By Visit 22 (Week 26)	5 (26.3) 4 (26.7)
Platelet response by IWG definition^f^, n (%)	
By Visit 4 (Day 8) By Visit 22 (Week 26)	9 (47.4) 9 (60.0)

*CI* confidence interval, *IQR* interquartile range, *IWG* International Working Group, *PC* platelet count, *SD* standard deviation

^a^The cumulative number of weeks in which the PC is ≥ 50 × 10^9^/L during 26 weeks of treatment, in the absence of rescue therapy

^b^PC ≥ 50 × 10^9^/L at Visit 4 (Day 8) in the absence of rescue therapy before or on Visit 4 (Day 8)

^c^At least 6 weeks (i.e., ≥ 75%) with PC ≥ 50 × 10^9^/L during the last 8 weeks of the 26-week treatment period, in the absence of rescue therapy

^d^The maximum number of weeks of platelet response (PC ≥ 50 × 10^9^/L) for each patient, in the absence of rescue therapy

^e^PC ≥ 100 × 10^9^/L and absence of bleeding or rescue therapy at each analysis visit

^f^PC ≥ 30 × 10^9^/L, at least a twofold increase in baseline PC, and absence of bleeding or rescue therapy at each analysis visit

Durable platelet response (defined as 6 out of 8 weekly PCs ≥ 50 × 10^9^/L during the last 8 weeks of treatment over the 26-week core phase treatment period, in the absence of rescue therapy) was 42.1% (95% CI 20.3–66.5). The mean maximum duration of continuous platelet response (defined as the number of consecutive weeks with PC ≥ 50 × 10^9^/L, in the absence of rescue therapy) was 7.6 weeks (SD: 6.9). Complete response by IWG definition (defined as PC ≥ 100 × 10^9^/L and absence of bleeding or rescue therapy at each analysis visit) was achieved in 5 of 19 patients (26.3%) by Visit 4 (Day 8) and in 4 of 15 patients (26.7%) by Visit 22 (Week 26). Platelet response by IWG definition (defined as PC ≥ 30 × 10^9^/L, at least a twofold increase in baseline PC and absence of bleeding or rescue therapy at each analysis visit) was also achieved in 9 of 19 patients (47.4%) by Visit 4 (Day 8) and in 9 of 15 patients (60.0%) by Visit 22 (Week 26).

**Fig. 3:**
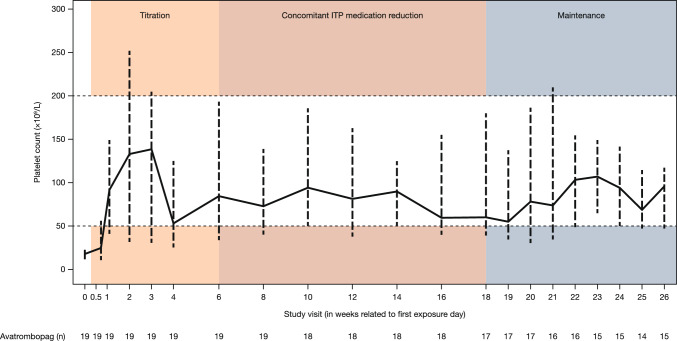
Median (IQR) platelet count by visit during the core phase (full analysis set). *ITP* immune thrombocytopenic purpura, *IQR* interquartile range

Median PC was within the target range of 50–200 × 10^9^/L during the core phase from Week 1 to Week 26 (Fig. 3). During the dose titration phase, median PC increased rapidly during the first 2 weeks, with a slight further increase in the third week, followed by a drop at Week 4 and subsequent stabilisation for the remainder of the study. The decrease in PC at Week 4 was due to dose adjustments in accordance with the dose adjustment guidelines.

The results for the subgroup analyses of the primary efficacy endpoint were generally consistent with the primary efficacy analysis. The mean cumulative number of weeks of platelet response was lower in males compared to females (7.9 vs 15.0 weeks), in splenectomised patients compared to non-splenectomised patients (10.0 vs 13.9 weeks), and in patients with baseline creatinine clearance of ≥ 30 to  < 60 mL/min compared to patients with baseline creatinine clearance of ≥ 60 to  < 90 mL/min and ≥ 90 mL/min (4.4 vs 15.2 and 13.9 weeks); however, the sample size of these subgroups was too small to allow for any conclusions about response rates.

Table 3 presents data for other efficacy endpoints not relating to platelet response. The proportion of patients who took at least one rescue therapy was 26.3% (95% CI 9.1–51.2). The proportion of patients who had a reduction in the number of total daily doses of concomitant ITP medications from baseline, without later increasing back to baseline or a higher dose, was 55.6% (95% CI 21.2–86.3). One patient received concomitant ITP medications at baseline and subsequently discontinued them (11.1%, 95% CI 0.3–48.2). Among the patients who experienced any bleeding event in the core phase, the majority were WHO Grade 1 bleeding events (14 patients). A WHO Grade 2 bleeding event (purpura) was experienced by one patient, and no patients experienced a WHO Grade 3 or 4 bleeding event.

**Table 3:** Efficacy: rescue therapy use, reduction/discontinuation of concomitant ITP medications and bleeding events (full analysis set)

	Avatrombopag (N = 19)
Rescue therapy use^a^, n (%) [95% CI]	5 (26.3) [9.1–51.2]
Reduction of concomitant ITP medications after baseline^b,c^, n (%) [95% CI]	5 (55.6) [21.2–86.3]
Discontinuation of concomitant ITP medications after baseline^b,d^, n (%) [95% CI]	1 (11.1) [0.3–48.2]
Bleeding events using the WHO bleeding scale^e^	
Grade 0	4
Grade 1	14
Grade 2	1
Grade 3 or 4	0

*CI* confidence internal, *ITP* immune thrombocytopenic purpura, *WHO* World Health Organization

^a^Patients with use of at least one rescue therapy after the first administration of study drug in the core phase

^b^The denominator is the number of patients who used concomitant ITP medications at baseline (n = 9)

^c^Patients with use of concomitant ITP medication at baseline and who had all post-baseline total daily doses for same medications (by preferred term) reduced without later increasing to the baseline or higher dose, or who had no use of concomitant ITP medication post-baseline during the core phase

^d^Patients with use of concomitant ITP medication at baseline and who discontinued all concomitant ITP medications before the last dose date of the core phase

^e^The highest grade a patient experienced during the core phase

### Safety

AEs in the core phase of the study are summarised in Table 4. A total of 18 (94.7%) patients experienced a TEAE, and 3 (15.8%) patients experienced a TEAE considered by investigators to be related to avatrombopag. Overall, 3 (15.8%) patients experienced an SAE and 3 (15.8%) patients experienced any TEAE classified as Common Terminology Criteria for Adverse Events (CTCAE) Grade ≥ 3 (two Grade 3, one Grade 4). One patient discontinued treatment due to an SAE of autoimmune hepatitis; however, the patient had a medical history of antiphospholipid syndrome. The most frequently reported TEAEs were COVID-19 and upper respiratory tract infection (3 patients, 15.8% each), followed by nasopharyngitis, nausea and urticaria (2 patients, 10.5% each). Regarding AESI, one patient experienced a bleeding event (heavy menstrual bleeding) that was CTCAE Grade 3 and resolved 11 days after the onset. This was not considered to be related to avatrombopag. There were no TEEs and no deaths reported.

**Table 4:** Overview of AEs (safety analysis set)

Patients with events, n (%)^a^	Avatrombopag (N = 19)
TEAEs	18 (94.7)
Treatment-related TEAEs^b^	3 (15.8)
Serious TEAEs	3 (15.8)
Treatment-related serious TEAEs	0
Most frequent (≥ 10%) TEAEs by preferred term, *n* (%)	
COVID-19	3 (15.8)
Upper respiratory tract infection	3 (15.8)
Nasopharyngitis	2 (10.5)
Nausea	2 (10.5)
Urticaria	2 (10.5)
TEAEs by maximum severity	
Grade 1	6 (31.6)
Grade 2	9 (47.4)
Grade 3	2 (10.5)
Grade 4	1 (5.3)
Grade 5	0
AESIs	1 (5.3)
Thromboembolic events	0
Bleeding events (CTCAE Grades 3 and 4)	1 (5.3)
Treatment-related AESIs	0
Deaths	0

*AE* adverse event, *AESI* adverse event of special interest, *CTCAE* Common Terminology Criteria for Adverse Events, *MedDRA* Medical Dictionary for Regulatory Activities, *TEAE* treatment-emergent adverse event

^a^AEs were classified using MedDRA version 26.1. If multiple AEs occurred in the same patient, the patient was counted once in the System Organ Class or Preferred Term. Patients with multiple grades were counted once in the maximum grade

^b^The treatment-related TEAEs were leukocytosis, palpitations, blood pressure increased, headache and urticaria

During the core phase, there were no substantial changes in mean values for haematology parameters (other than PC) and clinical chemistry parameters from baseline to Week 26. TEAEs related to abnormal laboratory values were reported in 3 patients, including increased blood lactate dehydrogenase (considered not related to avatrombopag). No relevant or treatment-related trends of change in vital signs (pulse rate, and systolic and diastolic blood pressure) of physician examination findings were noted in the core phase following study drug administration.

### PK and PD

Previously developed population PK and population PK/PD models which adequately described the data from this study, indicating there were no major differences between Japanese patients and the ‘legacy’ patient populations from all adult studies of avatrombopag to date (healthy subjects, patients with CLD, patients with ITP and patients with CIT).

The Japanese patient population in this study had a smaller average body weight than the legacy dataset (median weight of 61.7 kg compared to 74 kg). As such, the expectation was that Japanese subjects would be on the higher end of the exposures as compared to their legacy dataset counterparts. Comparison of the maximal concentration at steady-state (Cmax_ss_) from simulation of the legacy dataset (4000 simulated individuals treated with 20 mg) to Japanese individuals treated with 20 mg indicated Japanese patients had a higher Cmax_ss_ with a geometric mean (geometric coefficient of variation [CV]) of 263 (37.2) ng/mL compared to 165 (61.3) ng/mL of the legacy dataset. Similarly, area under the concentration time curve at steady-state (AUC_ss_) was higher for Japanese patients with a geometric mean (geometric CV) of 4991 (35) ng × h/mL compared to 3277 (62.2) ng × h/mL. The data were congruent with the expectation that Japanese patients would on average have higher geometric mean exposure estimates compared to the legacy dataset, likely due to their smaller average body weight.

## Discussion

In this study of avatrombopag in Japanese adults with primary chronic ITP refractory to other treatments, the primary efficacy endpoint was met, with a mean cumulative number of weeks with a PC ≥ 50×10^9^/L of 13.5 weeks (95% CI 9.1–17.8) during the 26-week core phase (in the absence of rescue therapy). The key secondary endpoint was also met, with 63.2% of patients achieving a PC ≥ 50×10^9^/L at Visit 4 (Day 8). These data support the overall efficacy of avatrombopag in demonstrating a rapid and durable platelet response in Japanese patients with chronic ITP.

These efficacy findings are broadly comparable to those from other global and Chinese phase 3 studies of avatrombopag in adult patients with chronic ITP. In the global phase 3 study, the median cumulative number of weeks with a PC ≥ 50 × 10^9^/L was 12.4 weeks in the avatrombopag group [26], compared to a median of 16.6 weeks in the current study. The response rate at Day 8 (key secondary endpoint) was 63.2% in the current study, 65.6% in the global phase 3 study [26], and 72.9% in the Chinese phase 3 study [27]. The durable platelet response rate in Japanese adult patients receiving avatrombopag was 42.1% in the current study, 34.4% in the global phase 3 study [26] and 43.8% in the Chinese phase 3 study [27]. Collectively, these phase 3 data demonstrate the consistent efficacy of avatrombopag across different populations of adults with primary chronic ITP.

Furthermore, the safety profile of avatrombopag in the current study was generally consistent with previous studies. There were no new or unexpected safety findings during the core phase of this study. In this phase, the most common TEAEs were COVID-19 infection, upper respiratory tract infection, nasopharyngitis, nausea and urticaria. No deaths or TEEs were reported, and the majority of TEAEs were Grade 1 or 2. One patient experienced a bleeding event (heavy menstrual bleeding) during the core phase that was CTCAE Grade 3 and not considered to be related to avatrombopag. In comparison, in the global phase 3 avatrombopag study, the most common AEs were headache and contusion, and the majority of bleeding events were WHO Grade 1, except for 3 patients in the avatrombopag group who experienced a Grade 2 or 3 bleeding event [26]. In the phase 3 study of avatrombopag in Chinese patients, the most common TEAEs in the avatrombopag group were upper respiratory tract infection, increased PC, petechia and headache [27]. Bleeding events occurred in 70.8% of patients in the avatrombopag group and 88.5% of patients in the placebo group, none of which were WHO Grade 3 or 4 [27]. While headache was not one of the most common AEs in the current study, it was reported as a treatment-related AE. Importantly, no significant hepatotoxicity was reported in the current study, in alignment with previous studies [26, 27]. There was no major difference in PK or PD between Japanese patients in this study and other populations in the ‘legacy’ dataset.

The efficacy and safety results of this study are broadly similar to those from other phase 3 studies in Japanese patients for the TPO-RAs romiplostim and eltrombopag, with the notable exception of hepatotoxicity being reported for eltrombopag [18, 19]. In a phase 3 study of romiplostim in adult Japanese patients with chronic ITP (N = 34), the median number of weeks with a PC ≥ 50 × 10^9^/L (primary efficacy endpoint) was 11 weeks (lower quartile 9, upper quartile 12) [12], which is somewhat lower than the median 16.6 weeks observed in the current study. In a phase 3 study of eltrombopag in Japanese patients with chronic ITP (N = 23, 65.2% female), the proportion of patients with a PC ≥ 50 × 10^9^/L at Week 6 at the end of the 6-week double-blind phase (primary endpoint) was 60% in the eltrombopag treatment group [10]. In the romiplostim study, the most common AEs included nasopharyngitis and headache [12]. There was one bleeding event in the romiplostim treatment group that was ≥ Grade 3 [12]. Nasopharyngitis and alanine aminotransferase increase were the most frequently reported AEs in the phase 3 eltrombopag study [10], with nasopharyngitis and headache being the most frequent AEs in a phase 3 open-label extension study of eltrombopag [11]. While nasopharyngitis was also commonly reported in the current avatrombopag study, and headache was commonly reported in previous phase 3 avatrombopag studies [26, 27], there were no substantial changes in laboratory values for haematology and clinical chemistry parameters. Notably, eltrombopag can cause severe hepatotoxicity, which might be life-threatening, and requires additional liver monitoring for all patients, as described in the box warning [18, 19]. Avatrombopag is associated with no significant hepatotoxicity [25, 26], and as such, there is no requirement for additional liver monitoring in patients with adequate hepatic function [29].

There are several notable differences in the populations included in the current study compared with these in the romiplostim and eltrombopag studies. Patients in the current study may have used other TPO-RAs prior to the study (although they were not permitted during the study), while the romiplostim and eltrombopag phase 3 studies did not include prior TPO-RA use as the approval dates for both romiplostim (first approved in Japan in 2011) and eltrombopag (first approved in Japan in 2010) were during or after the study periods [10–12, 15, 16]. Of the 19 patients enrolled in the current study, 15 had taken eltrombopag previously and 2 patients had taken romiplostim previously (both patients treated with romiplostim had also taken eltrombopag). While avatrombopag has previously been demonstrated to be effective in patients who have not been treated with any prior TPO-RAs [34], the data from the current study add to a growing body of evidence regarding the efficacy of avatrombopag in patients switching from another TPO-RA [23, 35]. Japanese treatment guidelines for ITP were revised in 2019, with TPO-RA treatment being repositioned from third-line treatment to second-line treatment [7], which aligns with guidelines from the American Society of Hematology and the International Consensus Report [6, 36]. This change in guidelines reflects how TPO-RAs have become more established as a treatment option for Japanese patients with chronic ITP, due to accumulated experience and evidence about their long-term efficacy and safety [7].

The results of this study should be interpreted in light of some limitations. The primary limitation of this study is the small sample size. Particularly, the sample size of the subgroups was too small to allow for any conclusions about response rates and, therefore, further studies/analyses may be warranted in order to investigate the potential impact of certain factors (e.g., concomitant ITP medications) on avatrombopag efficacy and safety. Additionally, the observation period was short, however, the extension phase will provide further data on longer term efficacy and safety. There was a relatively high proportion of women in this study: 78.9% compared with 63.6% in the phase 3 romiplostim study [12]. However, Japanese and global data do indicate there is a slightly higher incidence of ITP in females [3, 37, 38]. Future long-term and real-world evidence will inform the profile of avatrombopag in populations not studied in this trial (e.g., TEE history, malignancies). Finally, health-related quality of life (HRQoL) was not assessed in this study. While a previous study indicated that avatrombopag is effective in real-world populations and may improve HRQoL through improved clinical outcomes, treatment satisfaction, and adherence [39]*,* future studies focusing on the Asian population may benefit from incorporating instruments to assess HRQoL.

In conclusion, avatrombopag, an oral TPO-RA with no restrictions on meal composition, induced a rapid and sustained platelet response in Japanese adult patients with primary chronic ITP who are refractory to other treatments, and was well-tolerated in these patients. These data add to the body of evidence which supports the benefits of using avatrombopag to treat primary chronic ITP in different populations of adults, including those of different ethnicities, and highlight that there is no need for different or adjusted doses in the Japanese population compared with populations previously studied.

## Supplementary Information

Below is the link to the electronic supplementary material.

Supplementary file1 (DOCX 39 KB)

## Data Availability

The datasets analysed in the current study are available from the corresponding author on reasonable request. Sobi is committed to responsible and ethical sharing of data on the participant level and summary data for medicines and indications approved by the European Medicines Agency and/or Food and Drug Administration, while protecting individual participant integrity and compliance with applicable legislation. Data access will be granted in response to qualified research requests. All requests are evaluated by a cross‐functional panel of experts within Sobi and a decision on sharing will be based on the scientific merit and feasibility of the research proposal, maintenance of personal integrity and commitment to publication of the results. To request access to study data, a data sharing request form (available on http://www.sobi.com) should be sent to medical.info@sobi.com. Further information on Sobi’s data sharing policy and process for requesting access can be found at: https://www.sobi.com/en/policies.
