# Correction: A phase 3 study of the efficacy and safety of avatrombopag in Japanese adults with chronic immune thrombocytopenia

**DOI:** 10.1007/s12185-025-04054-5

**Published:** 2025-08-19

**Authors:** Hiroki Yamaguchi, Masaki Iino, Shugo Kowata, Ryusuke Yamamoto, Jun Yamanouchi, Yutaka Imamura, Keita Kirito, Kenji Yokoyama, Tomoki Ito, Tatsunori Ishikawa, Motoaki Shiratsuchi, Yoshiaki Tomiyama, Harumi Kamiya, Jessica Zhang, Brian D. Jamieson

**Affiliations:** 1 Department of Hematology, Nippon Medical School Hospital, Tokyo, Japan; 2 Department of Hematology and Hematopoietic Stem Cell Transplantation, Yamanashi Prefectural Central Hospital, Yamanashi, Japan; 3 Division of Hematology and Oncology, Department of Internal Medicine, Iwate Medical University, Iwate, Japan; 4 Department of Hematology, Kobe City Medical Center General Hospital, Hyogo, Japan; 5 Division of Blood Transfusion and Cell Therapy, Ehime University Hospital, Ehime, Japan; 6 Division of Hematology, St. Mary’s Hospital, Fukuoka, Japan; 7 University of Yamanashi, Yamanashi, Japan; 8 Tokai University Hachioji Hospital, Tokyo, Japan; 9 Kansai Medical University, Osaka, Japan; 10 Chugoku Central Hospital, Hiroshima, Japan; 11 Aso Iizuka Hospital, Fukuoka, Japan; 12 Department of Blood Transfusion, Osaka University Hospital, Osaka, Japan; 13 Sobi, K.K., Tokyo, Japan; 14 Sobi, Inc, Morrisville, NC USA

**Correction: International Journal of Hematology** 10.1007/s12185-025-04001-4

The article ‘A phase 3 study of the efficacy and safety of avatrombopag in Japanese adults with chronic immune thrombocytopenia’, written by Hiroki Yamaguchi, Masaki Iino, Shugo Kowata, Ryusuke Yamamoto, Jun Yamanouchi, Yutaka Imamura, Keita Kirito, Kenji Yokoyama, Tomoki Ito, Tatsunori Ishikawa, Motoaki Shiratsuchi, Yoshiaki Tomiyama, Harumi Kamiya, Jessica Zhang, Brian D. Jamieson, was originally published under exclusive license to Japanese Society of Hematology. As a result of the subsequent decision to publish the article under the open access model, the article’s copyright notice was changed on 25 July 2025 to © The Author(s) 2025 and the article is now distributed under a Creative Commons Attribution 4.0 International License, which permits use, sharing, adaptation, distribution and reproduction in any medium or format, as long as you give appropriate credit to the original author(s) and the source, provide a link to the Creative Commons licence, and indicate if changes were made. The images or other third party material in this article are included in the article's Creative Commons licence, unless indicated otherwise in a credit line to the material. If material is not included in the article's Creative Commons licence and your intended use is not permitted by statutory regulation or exceeds the permitted use, you will need to obtain permission directly from the copyright holder. To view a copy of this licence, visit http://creativecommons.org/licenses/by/4.0/.

