# Supplementary material for: A phase 3 study of the efficacy and safety of avatrombopag in Japanese adults with chronic immune thrombocytopenia
Source: Int J Hematol. 2025 May 20;122(4):521–32. doi: 10.1007/s12185-025-04001-4 (PMC12476303; doi:10.1007/s12185-025-04001-4)
Supplement: Supplementary file 1 — Supplementary file1 (DOCX 39 KB) [file 12185_2025_4001_MOESM1_ESM.docx]

# SUPPLEMENTARY MATERIALS

Supplementary Table S1 Avatrombopag dose adjustments

| Dose^a^ | Level |
| --- | --- |
| 40 mg once daily | 6 |
| 40 mg 3 times weekly AND 20 mg on the 4 remaining days of each week | 5 |
| 20 mg once daily | 4 |
| 20 mg 3 times weekly^b^ | 3 |
| 20 mg twice weekly OR 40 mg once weekly^c^ | 2 |
| 20 mg once weekly^d^ | 1 |
| **Platelet count (x10^9^/L)** | **Dose adjustment or action** |
| < 50 after at least 2 weeks of avatrombopag | - Increase one dose level - Wait 2 weeks to assess the effects of this regimen and any subsequent dose adjustments |
| ≥ 50 to < 200 | - Stay on the current dose level |
| ≥ 200 to ≤ 400 | - Decrease one dose level - Wait 2 weeks to assess the effects of this regimen and any subsequent dose adjustments |
| > 400 | - Stop avatrombopag - Increase platelet monitoring to twice weekly - When platelet count was  < 150×10^9^/L, decrease  1 dose level and reinitiate therapy |
| < 50 after 4 weeks of 40 mg avatrombopag once daily | - Discontinue avatrombopag |
| > 400 after 2 weeks of 20 mg avatrombopag weekly | - Discontinue avatrombopag |

^a^Patients taking avatrombopag less frequently than once daily took the medication in a consistent manner from week to week

^b^Dose Level 3: three non-consecutive days a week (e.g., Monday, Wednesday and Friday)

^c^Dose Level 2: two non-consecutive days a week (e.g., Monday and Friday)

^d^Dose Level 1: the same day each week (e.g., Monday)
